# Effect of hardening and sealing on color of chemically colored stainless steel

**DOI:** 10.1038/s41598-020-70359-6

**Published:** 2020-08-11

**Authors:** Keming Ji, Hongbin Ju, Jiayao Xun, Yuan Su, Kan Zhang, Ping Liu, Yongqiang Xue

**Affiliations:** 1 Institute of Coal Chemistry, Chinese Academy of Sciences, Taiyuan, 030001 China; 2 Taiyuan University of Technology, Taiyuan, 030024 China; 3 China Research Institute of Daily Chemistry Co., Ltd, Taiyuan, 030001 China; 4 China University of Petroleum, Beijing, 102249 China

**Keywords:** Chemistry, Engineering

## Abstract

A light reflection device combined with visible light spectrophotometer was used to measure the reflectance–wavelength curves of colored stainless steel after coloring, hardening and sealing. After these processes, the coordinate in CIE 1931 color coordinate system and brightness of samples were confirmed by chromatometry method, and the effects of hardening and sealing processes on color were determined. In hardening process, the thickness of coloring film increases significantly, the peaks of reflectance–wavelength curves move to the long-wave region, the hue of samples changes clockwise in CIE 1931 coordinate diagram, and the trend of saturation in hardening process is consistent with the saturation of the sample having larger potential difference. In sealing process, the thickness of film increases slightly, the peaks of reflectance–wavelength curves move to the long-wave region, the reflectivity and brightness of samples increase, the hue of samples moves clockwise in CIE 1931 coordinate diagram, and the saturation of some samples decreases.

## Introduction

Stainless steel has the advantages of high temperature resistance, corrosion resistance, long service life, 100% recyclable and harmless to human health. By 2019, the output of stainless steel in the world has reached 52.21 million tons, becoming one of the most important alloy materials. As a kind of decoration material, it needs bright-coloured and diverse appearance. The stainless steel coloring process can well meet this requirement.

The coloring methods of stainless steel include chemical coloring^1–5^, electrochemical coloring^6,7^, physical vapor deposition^8,9^, high temperature oxidation, laser pulse^10–12^, etc. Among them, the chemical coloring method, also known as International Nickel Corporation (INCO) coloring, is the most widely used method because of its bright colors^13^, excellent wear resistance^14^, which have been widely used. As decorative materials, color analysis on colored stainless steel samples is very important to their application. Musa et al.^13^ have measured the reflectivity of chemically colored stainless steel samples, their optical properties have been obtained, the results show that heating and covering by Si_3_N_4_ film could improve the absorption of sunlight. Vazquez-Santoyo et al.^15^ have measured wavelength-reflectance curves of colored stainless steel samples, and the colors of stainless steel samples have been expressed in Commission Internationale de l´Eclairage (International Commission on illumination, CIE) coordinate system by using chromatometry calculation. It can be seen that colors of stainless steel samples can be effectively expressed by reflectivity curves and CIE coordinate system. In our previous research^5^, we have applied the chromatometry principles on color analysis of different colored stainless steel samples, explored the brightness and the color cyclic change regularity of colored stainless steel with different color potential differences, and the color of samples are analyzed by CIE coordinate and munsell coordinate quantitatively.

In the practical production process, colored stainless steel samples should been hardening and sealing after coloring. These steps could increase the density, smoothness and firmness of coloring film, which is vital to improve the wear resistance and stain resistance of samples^16–18^.During these process, the color of sample will change^19,20^, however, the trend and the rule of color change is still lack of research. To understand the influence rule of hardening and sealing processes on colors of colored stainless steel is meaningful for the accurate coloring control of samples and the application of coloring technology.

In this paper, chromatometry analysis on colored stainless steel samples after coloring, hardening and sealing should be carried out, the effect of hardening and coloring processes on color will be researched, the change rules of optical and chromatometry properties of samples will be determined.

## Experimental

### Preparation of colored stainless steel^21^

SUS304(0Cr18Ni9) mirror polished steel was adopted as stainless steel samples, with the size as 30 mm × 70 mm × 0.5 mm. Samples were chemical degreasing and activation in 10%vol dilute sulphuric acid in 5 min, and then chemically colored by potential controlling method. The composition of chemical coloring solution is about CrO_3_ 265 g/L, H_2_SO_4_ 485 g/L, MnSO_4_ 4 g/L, ZnSO_4_ 5.5 g/L, (NH_4_)_6_Mo_7_O_24_ 7.5 g/L, and coloring temperature is 70 °C. A platinum electrode was used as a reference electrode to measure the relative potential between the stainless steel plate and the platinum electrode. The maximum potential was recorded, and as the relative potential dropped, the potential difference (the difference between the maximum relative potential and the instantaneous relative potential) gradually increased. When the potential difference reaches the target value, the sample was quickly taken out, rinsed by distilled water, and dried to determine the visible light reflectance of the sample. The coloring potential differences of samples prepared in this research are 10, 15, 20 and 25 mV, respectively.

**Figure 1:**
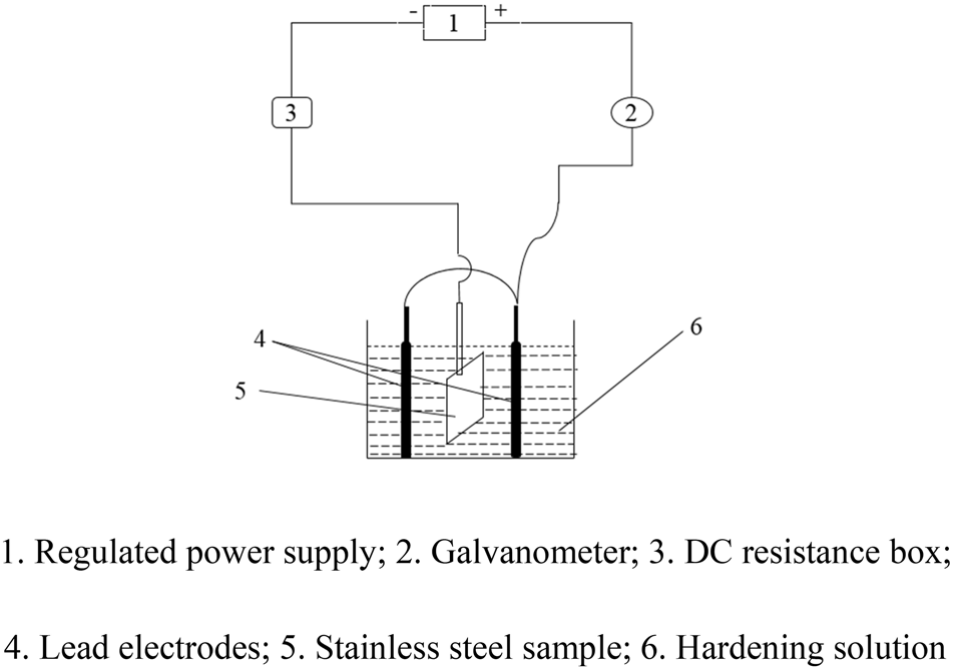
Diagram of hardening device.

Colored samples were hardened by electrochemical method. A colored stainless steel sample was used as cathode, and a lead plate as anode. The hardening device was connected as shown in Fig. 1, electrodes were placed in hardening solution. The coloring film can be hardened in 5 min at room temperature.

Composition of the hardening solution is CrO_3_ (250 g/L) and H_2_SO_4_ (2.5 g/L). The cathode current density is 2.33 mA/cm^2^. After the hardening operation, the sample was taken out, washed with distilled water, and dried to determine the visible light reflectance.

The hardened sample was placed in sealing solution, which contained 1.0% Na_2_SiO_3_, and boiled in 10 min. After the operation, the sample was taken out and washed with distilled water, then dried to determine the visible light reflectance.

### Determination of the reflectance at different wavelengths

**Figure 2:**
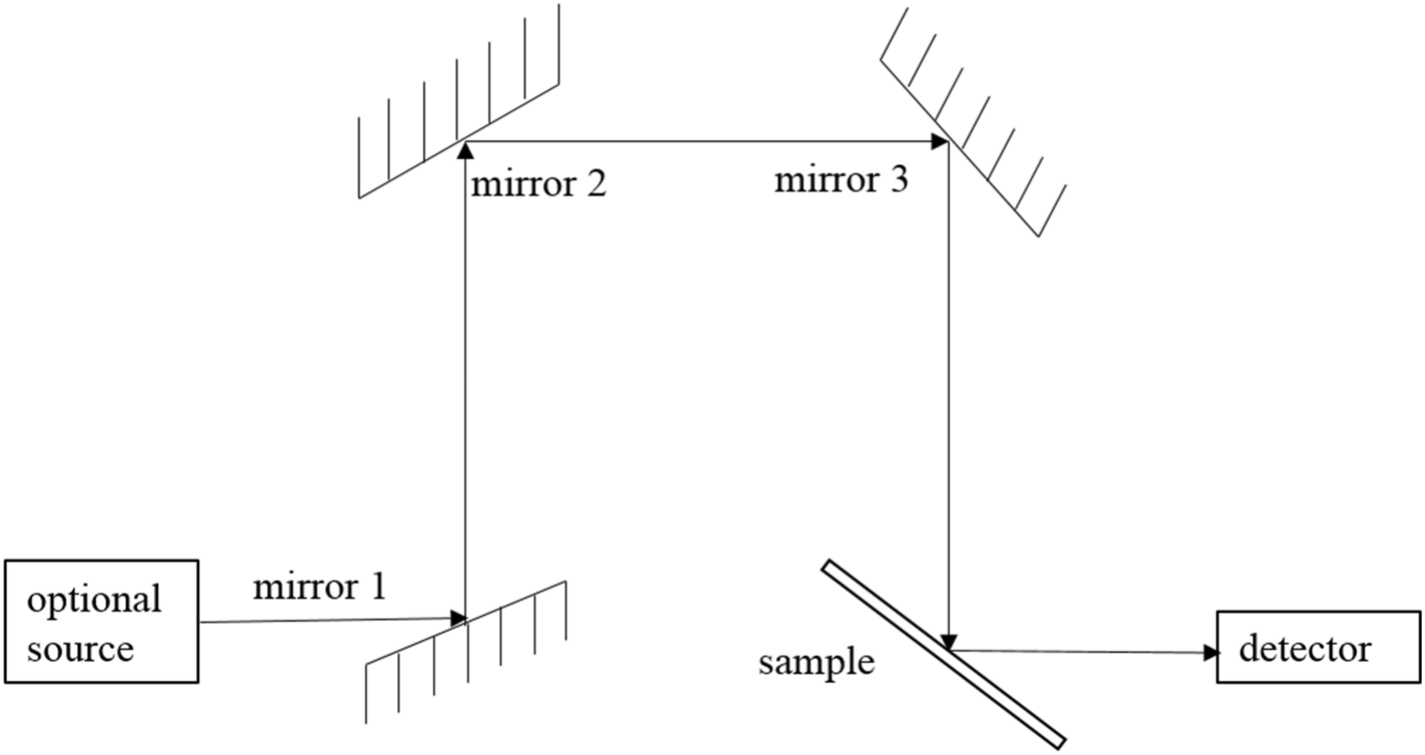
Schematic of the light-reflecting device.

A light-reflecting device was installed in the colorimetric chamber of the spectrophotometer shown in Fig. 2. Angles between each mirror or sample and the light path is 45°.

The reflectance at each wavelength of a sample before coloring was adjusted to 100% to get reflectance as the equal energy light source (white light). Then the reflectances of colored samples were detected at each 10 nm of wavelength.

## Results and discussion

### Reflectance of samples

**Figure 3:**
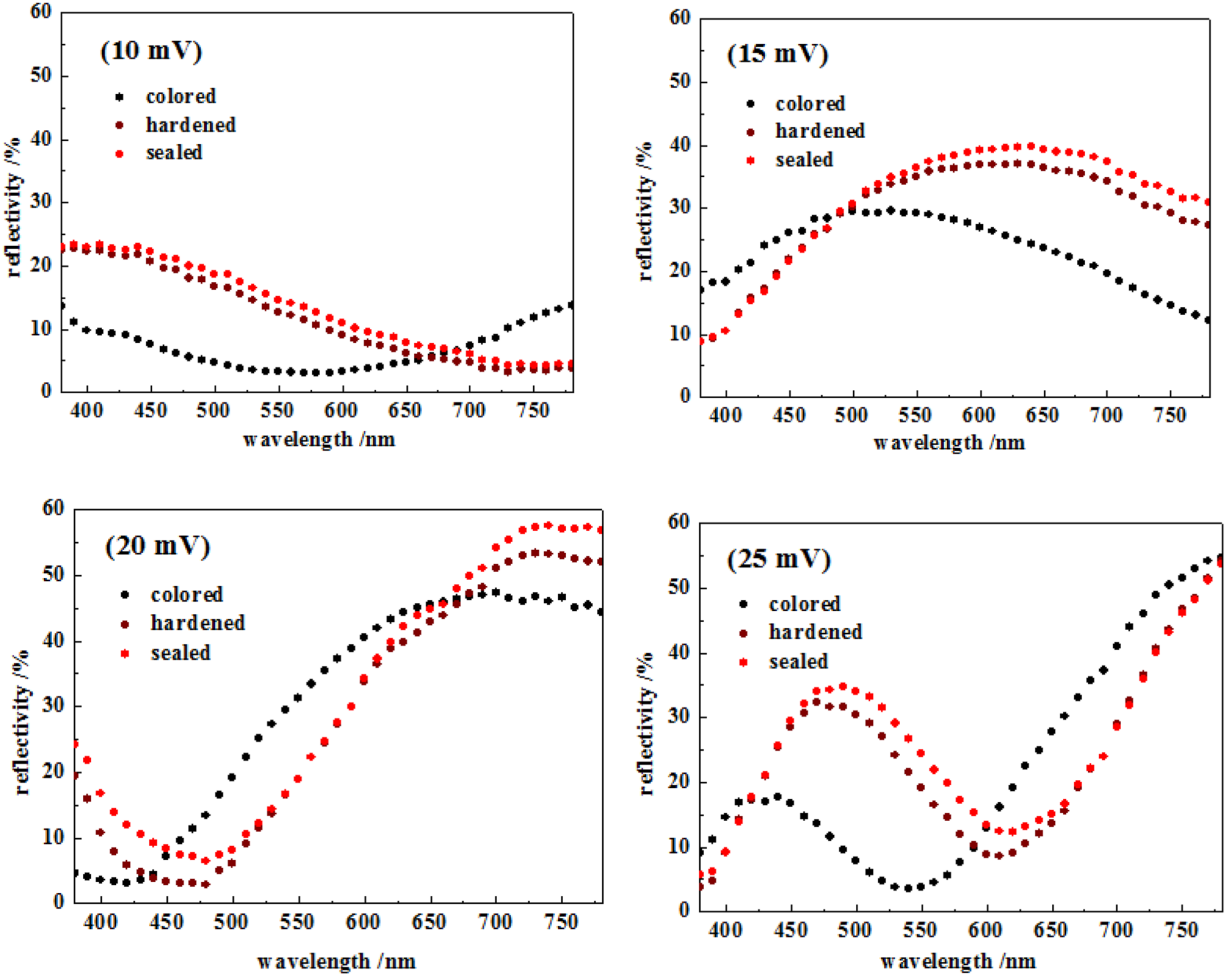
The reflectance–wavelength curves of samples.

According to the visible light reflectance of colored stainless steel samples, the reflectance–wavelength curves are shown in Fig. 3. It can be seen that samples with different potential differences have different reflectance–wavelength curves. There are significant differences in the position, height and number of peaks and troughs in the visible region of curves. Moreover, what is more important is that there are interesting changes and relations in the curves of colored, hardened and sealed samples with the same potential difference.

For colored sample with potential difference of 10 mV, there is no obvious peak in visible region, but only a trough at about 550 nm; after hardening, the trough moves significantly to the right to about 750 nm; after sealing, the trough continues to move slightly to the right, and the reflectances of the curve increase slightly at all wavelengths.

For colored sample with potential differences of 15 mV, there is no trough in the visible region, but only a peak at about 500 nm; after hardening, the curve shifts significantly to the right side and the peak moves to about 650 nm; after sealing, the position of the peak continues to move slightly to the right, and the reflectances above 550 nm increase.

For colored sample with potential difference of 20 mV, there is a trough at 400–450 nm and a peak at 650–700 nm in the visible region; after hardening, the curve moves to the right, the trough moves to 450–500 nm, and the peak moves above 700 nm; after sealing, the peak and the trough moves slightly to the right, and the reflectance of the range of peak and trough increases.

For colored sample with potential difference of 25 mV, there is a peak in the range of 400–450 nm, however, the peak is low and the reflectance is less than 20%; a trough places at about 550 nm; the reflectance continues to rise at higher wavelengths. After hardening, the curve moves to the right, the peak moves to the range of 450–500 nm, and the trough moves to about 600 nm. After sealing, the peak and the trough continue to move slightly to the right, and the reflectances of the area of peak and trough increase.

To summarize, the reflectance curves of samples with different potential differences at different wavelengths are similar to sine curves, which is consistent with the results of Musa et al.^13^ and Vazquez-Santoyo et al.^15^. The fluctuation of each curve is the result of light interference. Interference strengthening leads to peak, interference weakening lead to trough. According to characteristics of potential controlling coloring method^1^, coloring potential difference could control coloring process by replacing coloring time. By using AES, ELL and XPS, Xu et al.^22^ confirm that with the prolongation of coloring time, the thickness of coloring film increases correspondingly. The relation between the thickness of coloring film and the interference could be explained by the optical interference formula of film as follow:

1 $$ \delta  = 2e\sqrt {n_{2}^{2}  - n_{1}^{2} \sin ^{2} i}  = \left\{ {\begin{array}{*{20}l}    {k\lambda } \hfill & {k = 1,2,...\quad ({\text{strengthened)}}} \hfill  \\    {(2k + 1)\lambda /2} \hfill & {k = 0,1,...\quad ({\text{weakened}})} \hfill  \\   \end{array} } \right. $$

where $\delta$ is the optical path difference (nm), *e* is the thickness of the film (nm), *n*_*1*_ is the refractive index of air, *n*_*2*_ is the refractive index of the coloring film on colored stainless steel, *k* is the multiple of the wavelength (integer), $\lambda$ is the wavelength of incident light (nm), and *i* is the angle of incidence.

According to the strengthen condition which is $\delta$ = k $\lambda$ in Eq. (1), the wavelength corresponding to the strengthening region (peak) relates to the optical path difference (proportional to the thickness of the film, *e*). As the thickness of the film increases, the optical path difference increases correspondingly, and the wavelength of the interference strengthened also increases. Peaks move from the ultraviolet region to the short-wave region of visible light first, and then to the longer-wave side. The movement of peaks on interference curves to the longer-wave side due to the hardening and sealing process leads to the increase of the thickness of the coloring film^20^. As shown in Fig. 3, after the hardening and the sealing processes, peaks all move to the longer-wave side, and the curves of hardening samples move significantly, while the curves of sealing ones move less. It indicates that the thickness of the film increases more in hardening process, while less in sealing process. In addition, all of the reflectance curves of sealed samples slightly move up, which relates to the improvement of surface finishing and reflectance of samples.

### Colorimetry analysis of samples

The CIE 1931 chromatometry system is a system to describe the hue, brightness, and saturation of colors. Tristimulus values, chromatometry coordinates, and dominant wavelengths are included in this system. According to the reflectance–wavelength curves, these values of colors can be obtained.

#### Tristimulus values

In CIE 1931 chromatometry system, the X, Y, and Z primary values are used to match the spectrum stimuli of a color which are called the tristimulus values of this color. E is defined as the corresponding point of white light in the CIE coordinate system, the corresponding three stimulus values are respectively expressed as $\overline{x} (\lambda )$, $\overline{y} (\lambda )$, $\overline{z} (\lambda )$. In order to calculate the chromaticity coordinate of a color, it is necessary to calculate the tristimulus value of each color according to the spectral reflectance of each sample.

2 $$ X = k\sum\limits_{\lambda } {R\left( \lambda \right)S\left( \lambda \right)\overline{x} \left( \lambda \right)\Delta \lambda } $$

3 $$ Y = k\sum\limits_{\lambda } {R\left( \lambda \right)S\left( \lambda \right)\overline{y} \left( \lambda \right)\Delta \lambda } $$

4 $$ Z = k\sum\limits_{\lambda } {R\left( \lambda \right)S\left( \lambda \right)\overline{z} \left( \lambda \right)\Delta \lambda } $$

In these formulas, X, Y and Z are tristimulus values of a sample, respectively. R($\lambda$) is the reflectance of the sample at different wavelengths, S($\lambda$) is the relative spectral power distribution of light source at different wavelengths, $\overline{x} (\lambda )$, $\overline{y} (\lambda )$, $\overline{z} (\lambda )$ is tristimulus values of E at different wavelengths, the wavelength interval $\Delta \lambda$ = 10 nm, *k* is the adjustment factor, it is obtained by adjusting the Y value of the light source to 100, such as

5 $$ k = \frac{100}{{\sum\limits_{\lambda } {S\left( \lambda \right)\overline{y} \left( \lambda \right)\Delta \lambda } }} $$

The tristimulus values of samples are shown in Table 1.

**Table1:** Tristimulus values, chromaticity coordinates and principal wavelength of samples.

Coloring potential difference (mV)	State	X	Y	Z	x	y	Dominant wavelength (nm)
(Point E)		100	100	100	0.333	0.333	–
10	Colored	4.26	3.64	7.32	0.280	0.239	450
	Hardened	11.36	12.21	20.18	0.260	0.279	481
	Sealed	13.17	14.15	21.62	0.269	0.289	482
15	Colored	26.67	28.20	25.93	0.330	0.349	540
	Hardened	33.80	34.72	22.21	0.373	0.383	576
	Sealed	35.57	36.31	22.02	0.379	0.387	577
20	Colored	33.81	32.54	8.44	0.452	0.435	580
	Hardened	26.96	22.22	4.14	0.506	0.417	587
	Sealed	28.45	22.95	8.78	0.473	0.381	591
25	Colored	13.75	8.52	14.93	0.370	0.229	− 533
	Hardened	14.78	18.00	27.01	0.247	0.301	486
	Sealed	18.39	22.53	28.21	0.266	0.326	491

#### Brightness

**Figure 4:**
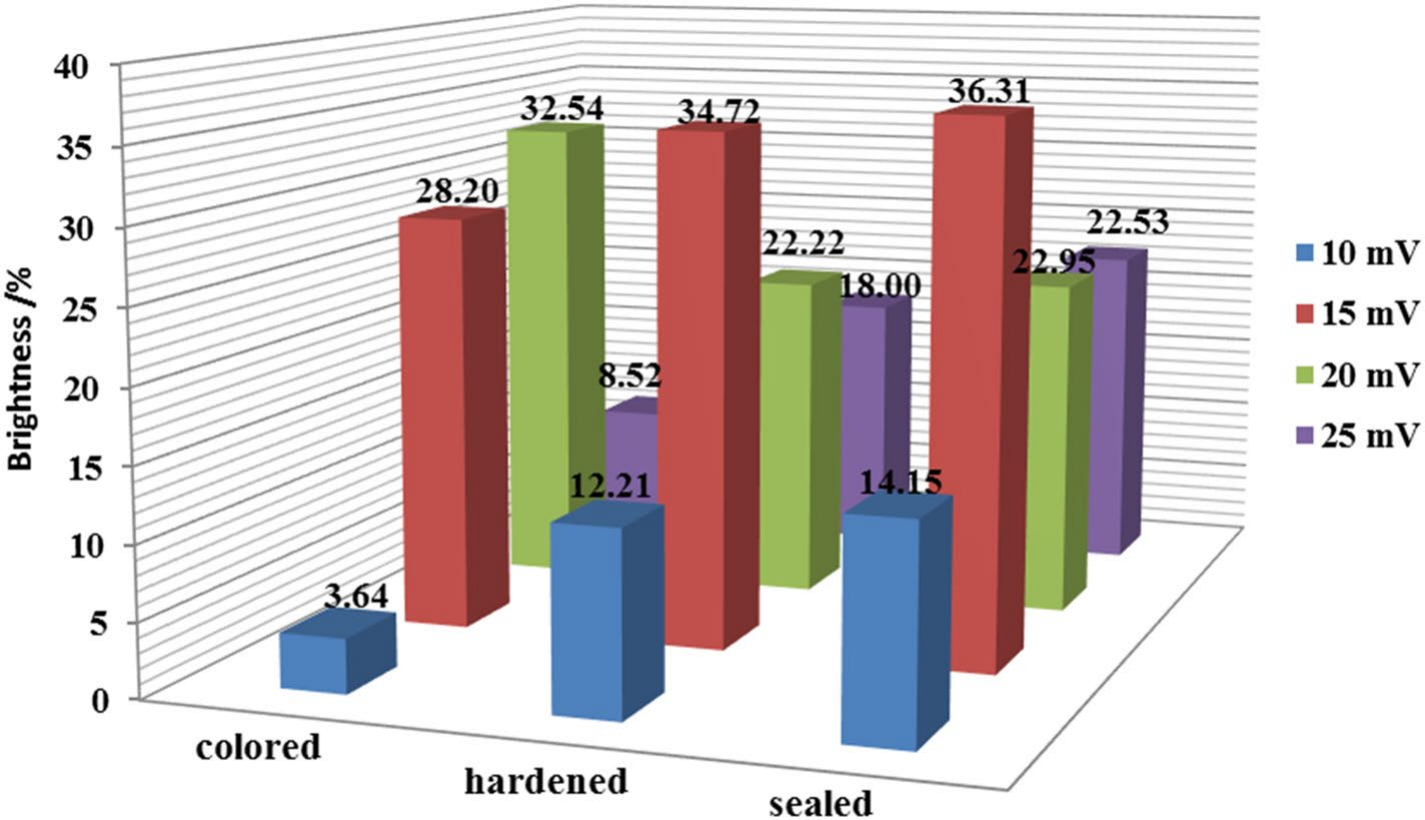
Brightness value of samples.

The Y value in the tristimulus value can represent the brightness of the sample, which is shown in Fig. 4. After hardening process, the brightness of samples with potential differences of 10 mV, 15 mV and 25 mV increases, while the brightness of sample with potential differences of 20 mV decreases. The change of brightness is consistent with the trend caused by the film thickness change.

After sealing process, the brightness with potential differences of 10 mV, 15 mV, 20 mV and 25 mV increases 1.94%, 1.59%, 0.73% and 4.53%, respectively. It can be seen that the brightness of sealed samples have an increasing trend, which dues to the increasing of the reflectance caused by the increase of the film density and the increase of the film flatness in sealing process ^20^.

#### Chromaticity coordinate

According to the following formula, the CIE 1931 coordinate, *x* and *y*, can be finally determined.

6 $$ x = \frac{X}{X + Y + Z} $$

7 $$ y = \frac{Y}{X + Y + Z} $$

The colorimetric coordinates of samples are shown in Table 1. The coordinate locus in the CIE 1931 coordinate diagram are drawn according with the coordinate of (x, y), which represents the colors.

**Figure 5:**
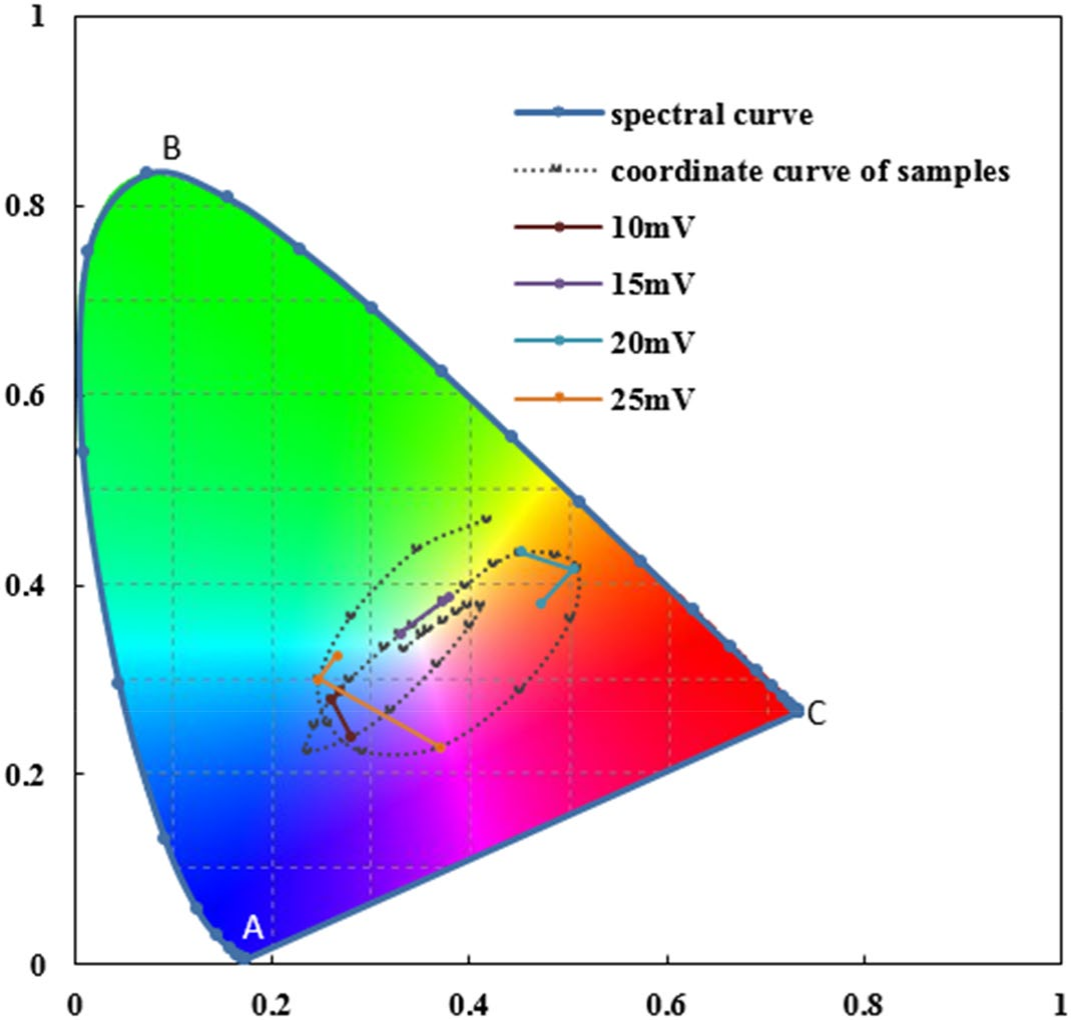
The coordinate track of samples after hardening and sealing in CIE 1931 diagram.

Figure 5 is CIE 1931 chromaticity diagram, which can intuitively represent the hue and saturation properties of a color. The point E is the equal energy point, corresponding to white light; the curve ABC is spectral curve, connecting coordinates of adjacent wavelength; straight line CA shows the color change from red, red purple to purple. In the chromaticity diagram, the ray emitted from point E in any direction is roughly in the same hue, and the farther the coordinate point is from point E, the greater the color saturation is, which means more colorful the sample is. In other words, the direction of the line between a point of a color and E can be used to represent the hue of the color, and the distance between these points can represent the saturation. The chromaticity coordinates of colored samples with potential differences in 0–30 mV are connected by a dotted line. Colored, hardened and sealed samples with coloring potential differences of 10 mV, 15 mV, 20 mV and 25 mV are joined with solid line.

It can be seen that after the hardening process of each sample, the hue significantly moves clockwise in the chromaticity diagram. Colors of hardened samples are almost on the curve of the connection of the samples with different coloring potential differences, indicating that the change rule of saturation of the hardening process is similar to that of increasing of coloring potential differences. Comparing with coordinates of hardened sample, coordinates of sealed samples also move clockwise, however, the distance is less than that of hardened ones. In addition, some of coordinates of sealed samples place at the inner side of the curve of samples with different potential differences, which indicates that the saturation of these samples decreases.

#### Dominate wavelength

According to the CIE1931 coordinate of samples in Fig. 5, the dominant wavelength of each sample can be obtained. This factor can easily represent the hue of a sample. The dominate wavelength values of each sample are shown in Table 1. It can be seen that the dominate wavelength of each sample changes from small to large after coloring, hardening and sealing. This is consistent with the change rule reflected in Fig. 5.

## Conclusion

The hardening and sealing processes can affect the optical and colorimetric properties of chemically colored stainless steels significantly. During the hardening process, the thickness of coloring film of colored stainless steel increases significantly, the reflectance–wavelength curve moves to the long-wave region, and the hue of sample changes clockwise in the CIE1931 coordinate diagram, the trend of saturation of hardened sample is consistent with the saturation of the sample having larger potential difference. In the process of sealing, the thickness of coloring film increases slightly, and the reflectance–wavelength curve moves to the longer wavelength side, the reflectance increases and the brightness rises, the hue of the sample changes clockwise in the CIE 1931 coordinate diagram, and the saturation of some sample decreases.
